# Case Report: Atypical femoral fractures in adult-onset hypophosphatasia treated with low-dose asfotase alfa and adjunctive teriparatide therapy

**DOI:** 10.3389/fendo.2026.1909920

**Published:** 2026-10-08

**Authors:** Yoon-Sok Chung, Kwang-Kyoun Kim, Gokul B. S., Ji-Seong Park

**Affiliations:** 1 Department of Endocrinology and Metabolism, Ajou University School of Medicine, Suwon, Republic of Korea; 2 Department of Orthopedic Surgery, Konyang University Hospital, Daejeon, Republic of Korea; 3 Department of Orthopedic Surgery, Sri Madhusudhan Sai Institute of Medical Sciences and Research, Karnataka, India

**Keywords:** adult-onset, asfotase alfa, atypical femur fracture, hypophosphatasia, teriparatide

## Abstract

Hypophosphatasia is a rare inherited metabolic bone disease caused by low activity of tissue-nonspecific alkaline phosphatase. Adult-onset hypophosphatasia is increasingly recognized, but fracture patterns and optimal management remain unclear. We report a 52-year-old woman who presented with progressive pain and bilateral atypical femoral fractures. She was diagnosed with hypophosphatasia based on low alkaline phosphatase, markedly elevated pyridoxal 5′-phosphate, a history of metatarsal fracture, premature tooth loss, and pathogenic ALPL variants. Low-dose asfotase alfa was started because standard-dose therapy was not feasible. After 6 months, callus formation was observed, and pain improved. However, 6 months later, the pain recurred, and radiographs showed a progressive fracture, suggesting insufficient fracture healing. Staged intramedullary fixation was performed for both femurs. Two perioperative femoral fractures occurred during the course of surgical management. Therefore, adjunctive teriparatide (20 µg daily for six months) was initiated while continuing low-dose asfotase alfa. At 5-year follow-up, radiographic union of all femoral fractures was achieved, and the patient remained ambulatory without assistance and had no fracture-related pain. This case shows that adult-onset hypophosphatasia can cause severe skeletal fragility and may require individualized multidisciplinary management, including biochemical diagnosis, long-term radiographic surveillance, cautious surgical planning, and selective use of medication.

## Introduction

Adult-onset hypophosphatasia (HPP) is an underrecognized cause of atypical femoral fractures and impaired fracture healing. Unlike osteoporosis, its pathophysiology is due to defective mineralization rather than low bone mass, creating unique diagnostic and therapeutic challenges (1). In adults, HPP commonly presents with musculoskeletal pain, recurrent or delayed-healing metatarsal stress fractures, and atypical subtrochanteric or femoral shaft fractures (1, 2). The disorder is caused by pathogenic variants in the gene encoding tissue-nonspecific alkaline phosphatase (TNSALP), an enzyme expressed on osteoblast surfaces that is essential for normal bone mineralization (3, 4).

TNSALP hydrolyzes substrates such as inorganic pyrophosphate (PPi) and pyridoxal 5′-phosphate (PLP). Reduced enzyme activity leads to extracellular accumulation of PPi, a potent inhibitor of hydroxyapatite formation, thereby impairing bone matrix mineralization (3). This results in reduced bone strength and increased susceptibility to insufficiency and atypical fractures. Delayed mineralization of fracture callus further contributes to impaired healing and delayed union (1, 3).

Asfotase alfa is a recombinant human TNSALP used as enzyme replacement therapy in HPP and has demonstrated improved skeletal mineralization and clinical outcomes, particularly in pediatric-onset disease (5, 6). However, evidence regarding its optimal dosing and effectiveness in adult-onset HPP remains limited. We report an adult-onset HPP case with bilateral atypical femoral fractures in which reduced-dose asfotase alfa produced early biochemical and radiographic improvement but did not prevent later fracture progression, and fracture union was achieved after staged fixation and adjunctive teriparatide therapy. Our case represents the first reported case of enzyme replacement therapy for adult HPP in South Korea.

## Case presentation

A 52-year-old woman presented to the outpatient clinic with right thigh pain that had developed two years earlier without a history of trauma. The pain occurred during walking and had progressively worsened. The patient was 155 cm tall and weighed 62 kg. Physical examination revealed poor dentition, tenderness over the right thigh, and a waddling gait.

Laboratory evaluation demonstrated markedly reduced alkaline phosphatase (ALP) levels of 11 IU/L (reference range, 35–104 IU/L), elevated pyridoxal 5′-phosphate (PLP) of 803.6 μg/L (20.0–125.0 μg/L), and mildly elevated serum calcium of 10.7 mg/dL (8.0–10.5 mg/dL). Inorganic phosphorus was 3.94 mg/dL (2.5–4.5 mg/dL), creatinine was 0.93 mg/dL (0.6–1.1 mg/dL), and 25-hydroxyvitamin D was 20.58 ng/mL (12–88 ng/mL). Serum N-telopeptide of type I collagen (NTX), serum procollagen type I N-terminal propeptide (P1NP), parathyroid hormone, and thyroid function tests were within normal limits.

Radiographs demonstrated an incomplete subtrochanteric fracture of the right femur. Bone scintigraphy revealed focal uptake in both femurs, the right first metatarsal, and the proximal phalanx of the left first toe. A lateral radiograph of the left femur also demonstrated an incomplete femoral shaft fracture (**Figure 1**). Dual-energy X-ray absorptiometry showed T-scores and Z-scores within normal limits.

**Figure 1:**
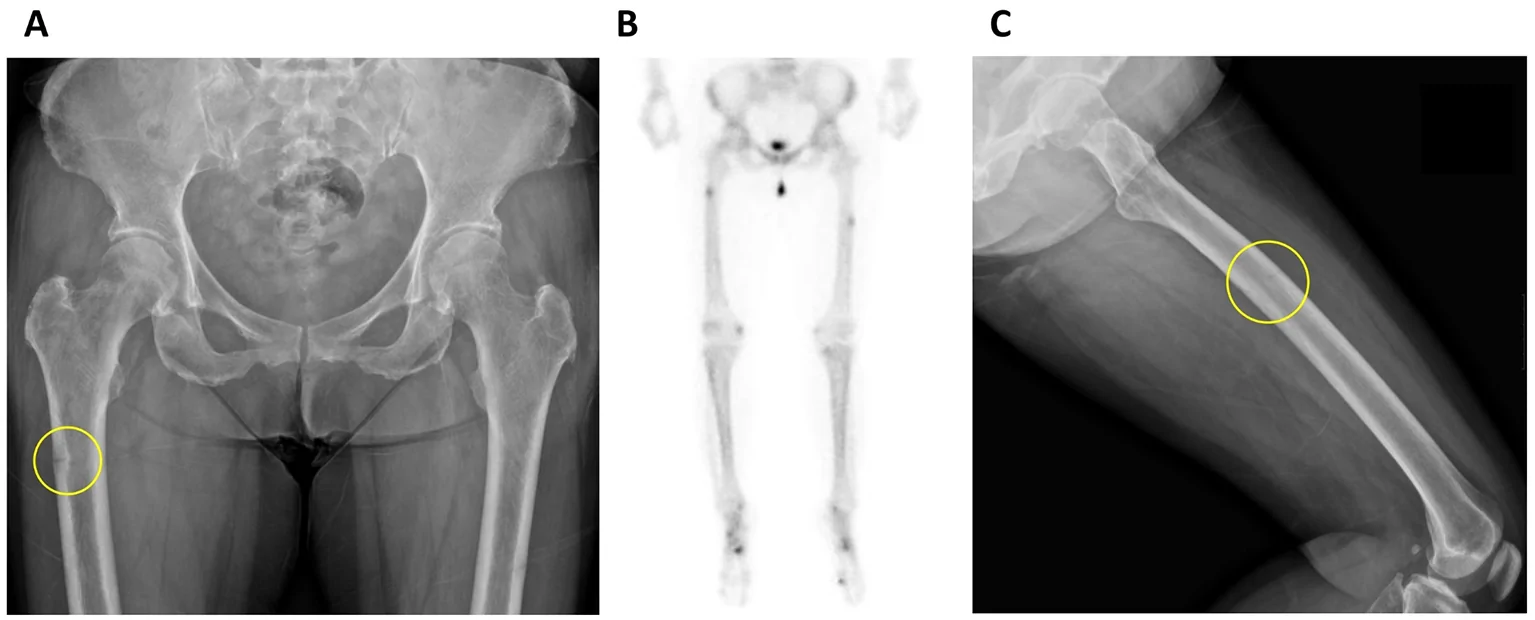
**(A)** Anteroposterior radiograph showing an incomplete subtrochanteric fracture of the right femur (yellow circle). **(B)** Whole-body bone scan demonstrating increased uptake in the bilateral femurs, right first metatarsal, and left proximal phalanx of the great toe. **(C)** Lateral radiograph showing an incomplete shaft fracture of the left femur (yellow circle).

Hypophosphatasia was suspected, and genetic testing supported the diagnosis with heterozygous ALPL mutations of c.29T>C (p.Ile10Thr) and c.382G>A (p.Val128Met). Both variants have been previously reported as pathogenic and are rare in the general population according to the Genome Aggregation Database and the Korean Reference Genome Database.

Initial treatment consisted of partial weight bearing, activity restriction, and low-dose asfotase alfa. Because asfotase alfa is not reimbursed for adult patients under the South Korean national health insurance system, a reduced-dose regimen was selected. She received a total of 12 doses over four months using a progressively tapered schedule. The patient received 40 mg subcutaneously once a week for seven weeks (0.65 mg/kg/week), followed by 40 mg once every two weeks for six weeks (0.32 mg/kg/week), and then 40 mg once every three weeks for six weeks (0.22 mg/kg/week). During treatment, ALP levels were 1,071 IU/L in the first phase, 138 IU/L in the second phase, and 63 IU/L in the third phase. PLP levels decreased from 803.6 µg/L before treatment to 403.3 µg/L during the second phase and 381.4 µg/L during the third phase. At 6 months after initiation of enzyme therapy, bridging callus formation was evident in both femurs, and the patient was able to ambulate without pain. Asfotase alfa was continued at 40 mg once every three weeks.

However, the initial clinical and radiographic response was not sustained. Six months later, right proximal thigh pain recurred, and radiographs showed bone resorption and lack of further fracture healing at the right femur. Pain also developed in the left thigh, with progression of the fracture line and persistent bone marrow edema on magnetic resonance imaging. Because both femoral fractures showed persistent pain, radiographic fracture progression, and delayed healing despite low-dose asfotase alfa, staged surgical fixation was planned.

Intramedullary long-nail fixation was first performed for the right subtrochanteric fracture. Postoperative radiographs revealed a peri-implant fracture at the proximal femoral screw insertion site. The fracture was managed conservatively, and the patient required walker assistance for ambulation. One month later, intramedullary nailing of the left femur was performed. The following day, she reported severe right knee pain. Radiographs revealed a new right distal femoral fracture, which was considered likely related to traction-table positioning during left femoral nailing in the setting of severe skeletal fragility (**Figure 2**). This additional perioperative fracture of the right distal femur was treated with distal femoral plate fixation. Because of delayed healing and recurrent fractures, daily subcutaneous teriparatide (20 μg) was initiated, while low-dose asfotase alfa was continued. Prior to initiation of teriparatide, the patient was informed that the use of teriparatide for fracture healing was off-label, and informed consent was obtained after explaining previously reported outcomes regarding fracture healing (8, 9). After initiation of teriparatide, bone turnover markers markedly increased. ALP increased to a peak of 1,042 IU/L and subsequently decreased to 337 IU/L at six months. P1NP increased to 338 ng/mL, and NTX to 371.9 nM bone collagen equivalents/mM creatinine. Urinary deoxypyridinoline (DPD) levels also increased to 34.9 nmol DPD/mmol creatinine. Teriparatide was discontinued after six months, once radiographic union and pain relief had been achieved. Low-dose asfotase alfa therapy was continued until 3 years (**Table 1**).

**Figure 2:**
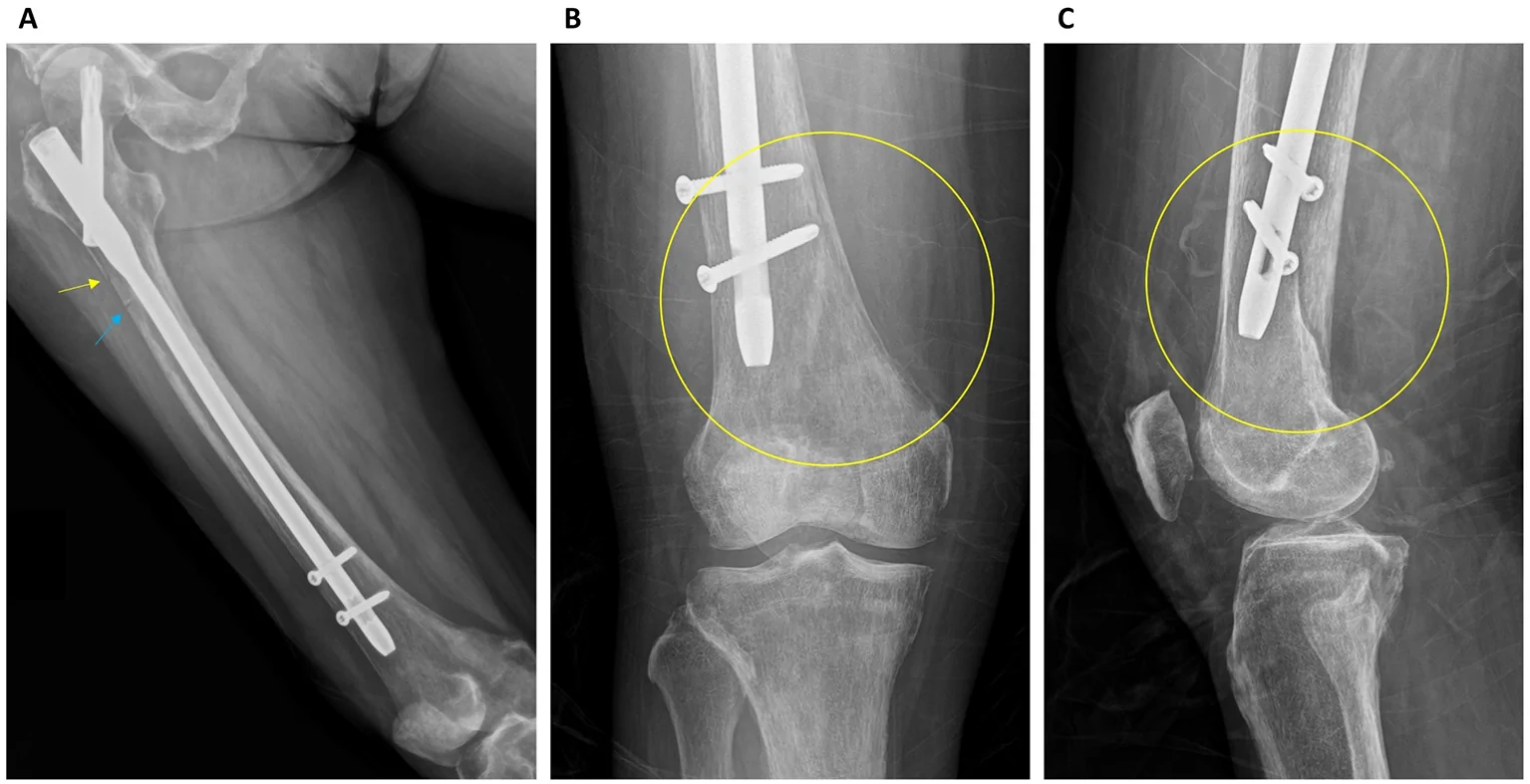
**(A)** Anteroposterior radiograph of the right femur after intramedullary nail fixation for an atypical femoral fracture. New perioperative fracture (yellow arrow) and initial atypical fracture (blue arrow) can be seen in the proximal area of the femur. **(B)** Anteroposterior radiograph of the right distal femur showing a newly developed fracture at the distal screw site (yellow circle). **(C)** Lateral radiograph of the right distal femur demonstrating the new fracture (yellow circle).

**Table 1:** Key laboratory results during the 5-year follow-up.

Follow-up (years)	Baseline	After asfotase alfa	1 year	1.5 years (with teriparatide)	2 years	3 years	4 years	5 years
ALP (U/L)	11	1,071	138	337	124	24	15	14
PLP (μg/L)	803	403	381	332	230	444	743	962

ALP, alkaline phosphatase; PLP, pyridoxal 5′-phosphate.

Five years after initial presentation, the patient remained pain-free and walked independently without support. Radiographs showed complete union of the right subtrochanteric, right distal femoral, and left femoral fractures (**Figure 3**).

**Figure 3:**
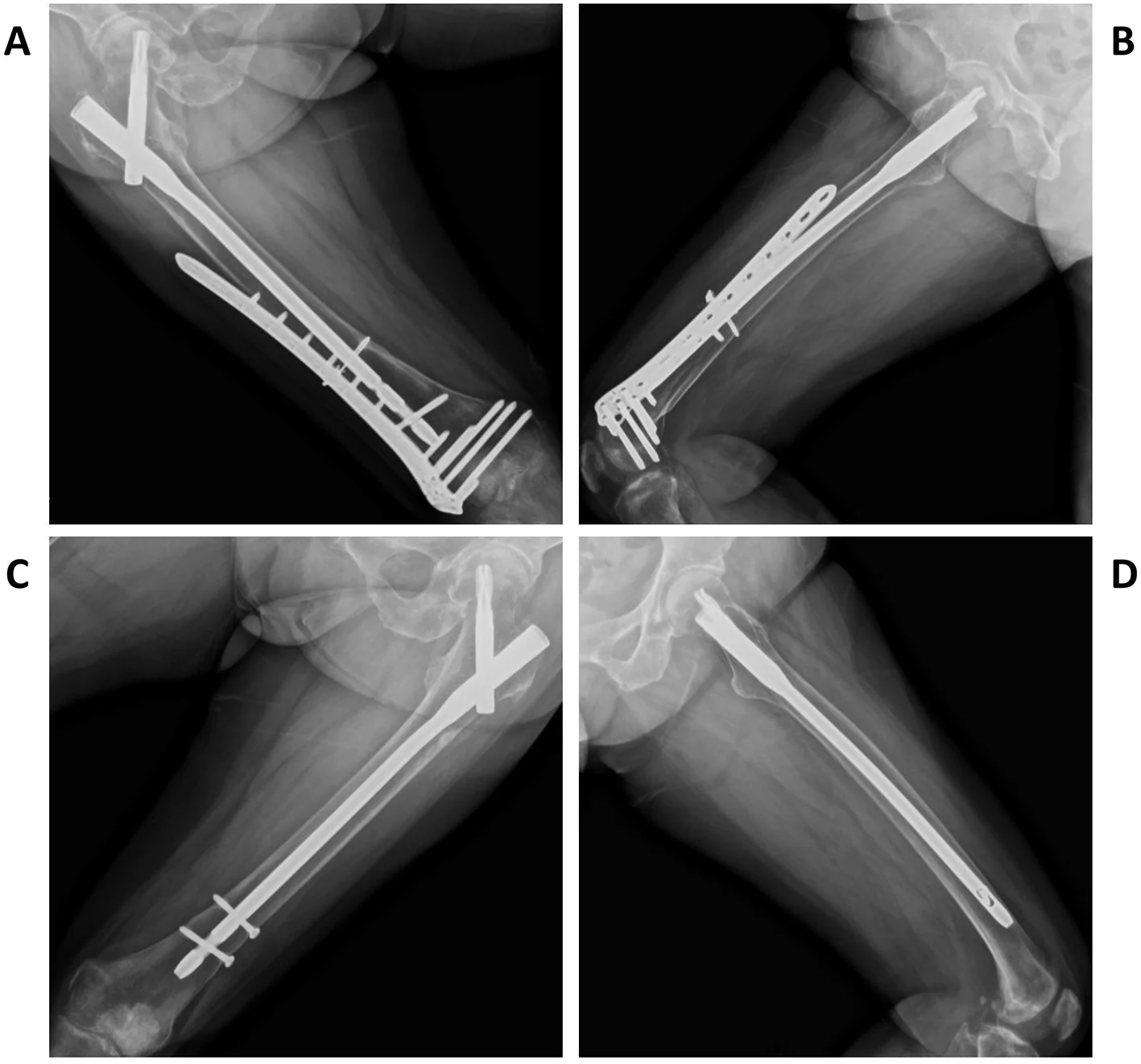
Final follow-up radiographs at 5 years. **(A)** Anteroposterior and **(B)** lateral radiographs of the left femur at 5-year follow-up since initial presentation showing a healed atypical femoral fracture. **(C)** Anteroposterior and **(D)** lateral radiographs of the right femur at 5-year follow-up since initial presentation, showing healed proximal atypical and distal femur fractures.

## Discussion

HPP is caused by loss-of-function mutations in the ALPL gene, which encodes TNSALP (4). Over 400 pathogenic variants have been described, accounting for the broad phenotypic spectrum (3). Disease severity depends on inheritance pattern and residual enzyme activity, with severe forms typically inherited in an autosomal recessive manner and milder adult-onset forms showing variable inheritance (7).

The finding of a mutation in the ALPL gene provided an additional level of confirmation. The patient had compound heterozygous ALPL mutations (c.29T>C [p.Ile10Thr] and c.382G>A [p.Val128Met]) previously reported in association with HPP (10). According to the American College of Medical Genetics and Genomics guidelines, both were classified as “likely pathogenic variants”. These genetic findings were consistent with the patient’s clinical presentation (phenotype) and mode of inheritance, confirming them as the causative etiology (“positive”) of hypophosphatasia.

Adult-onset HPP is characterized by persistently low ALP levels and atypical femoral fractures. However, hypophosphatasaemia may also occur in conditions such as hypothyroidism, Cushing syndrome, vitamin D intoxication, bisphosphonate exposure, and malignancy (1). Measurement of PLP is therefore particularly useful, as its elevation reflects TNSALP deficiency and helps distinguish HPP from secondary causes (2).

Atypical femoral fractures may also be associated with antiresorptive therapy, glucocorticoid use, malignancy-related treatments, or metabolic disorders such as hyperparathyroidism (11–14). In contrast, fractures in HPP result primarily from impaired mineralization due to PPi accumulation.

In this case, the patient experienced persistent pain and delayed fracture union during low-dose asfotase alfa treatment. However, complete bony union and long-term stability were successfully achieved through a multidisciplinary combination of surgical intervention, asfotase alfa, and teriparatide. Therefore, when managing atypical femoral fractures in adult-onset hypophosphatasia, clinicians should consider integrating surgical stabilization—a primary driver of fracture union—with sequential pharmacological interventions.

The surgical course in this patient highlights the extreme fragility of bone in adult HPP. Multiple perioperative fractures likely resulted from poor bone quality, stress concentration around implants, and vulnerability during positioning. In particular, when positioning the patient on a fracture table for hip fracture surgery, excessive torsional stress on the non-fractured limb must be strictly avoided, and protective adjuncts, such as prophylactic splinting, should be considered to prevent iatrogenic injury. These complications emphasize that operative fixation in patients with HPP warrants heightened caution and meticulous procedural planning.

Asfotase alfa was approved in 2015 for pediatric-onset HPP and later extended to all age groups in 2018 (5). The recommended dose is 6.0 mg/kg/week, given either as 2.0 mg/kg three times weekly or 1.0 mg/kg six times weekly. Treatment at this dose has been shown to normalize TNSALP substrate levels and improve functional outcomes in adults (15, 16). Dose–response studies indicate that lower doses may be insufficient to normalize PLP levels, whereas higher doses may suppress PPi, suggesting a narrow therapeutic window (16). Despite this, several reports have shown clinical and radiologic improvement even with substantially lower doses (as low as 0.1 mg/kg/week in one report) (17, 18). While low-dose asfotase alfa therapy cannot be generally advocated, this regimen was utilized in our patient due to socioeconomic limitations. Nonetheless, the recurrence of symptoms highlights the possibility that low-dose therapy was insufficient and emphasizes the need for concurrent surgical stabilization in atypical hip fractures. Further clinical studies are required to validate these findings.

Teriparatide, which has demonstrated favorable outcomes in adult HPP, was introduced to promote osteoporotic fracture healing (8, 9, 19–22). In our case, combination therapy with teriparatide and asfotase alfa resulted in increased bone turnover, successful fracture union, and sustained functional recovery. This suggests a potential complementary effect in which enzyme replacement addresses the mineralization defect and anabolic therapy enhances bone formation (1, 19).

In conclusion, adult HPP patients presenting with painful, progressive atypical femoral fractures may require a combined multidisciplinary approach comprising surgical fixation, asfotase alfa, and teriparatide if symptoms and radiographic progression continue despite enzyme replacement therapy. Furthermore, surgical intervention carries a substantial risk of iatrogenic fracture due to underlying bone fragility.

## Data Availability

The original contributions presented in the study are included in the article. Further inquiries can be directed to the corresponding author.
