# Supplementary material for: Case Report: Atypical femoral fractures in adult-onset hypophosphatasia treated with low-dose asfotase alfa and adjunctive teriparatide therapy
Source: Front Endocrinol (Lausanne). 2026 Oct 8;17:1909920. doi: 10.3389/fendo.2026.1909920 (PMC13660733; doi:10.3389/fendo.2026.1909920)
Supplement: Supplementary file 1 [file Table1.docx]

Supplementary Material

# 1 Supplementary Table

| **Timeline of clinical course** | | | | |
| --- | --- | --- | --- | --- |
| **Time point** | **Clinical event** | **Key findings** | **Medication** | **Outcome** |
| Sep 2020 | Initial presentation, diagnostic work-up, and initial treatment period | A 52-year-old woman presented with chronic right thigh pain without trauma.  Radiographs showed an incomplete atypical subtrochanteric fracture of the right femur and an incomplete shaft fracture of the left femur.  Laboratory results showed low alkaline phosphatase and elevated pyridoxal 5′-phosphate. Compound heterozygous ALPL variants were identified, and bone mineral density was within the normal range. | Low-dose asfotase alfa initiated | Patient was diagnosed with hypophosphatasia and atypical femur fracture. Partial weight bearing and activity restriction were initiated alongside medication. |
| May 2021 | Six months after initiation of asfotase alfa | Bridging callus formation was seen in both femurs. | Low-dose asfotase alfa | The patient was ambulatory without pain. |
| Nov 2021 | Right proximal thigh pain recurred, and left thigh pain developed. | Radiographs showed lack of further healing in the right femur. The left femoral fracture line progressed, with persistent bone marrow edema on magnetic resonance imaging. | Low-dose asfotase alfa | Staged surgical fixation of both femurs was planned. |
| Jan 2022 | Right femoral fracture fixation. | Incomplete atypical subtrochanteric fracture of the right femur. | Low-dose asfotase alfa | Closed reduction and internal fixation with intramedullary long-nail fixation was performed. A peri-implant fracture at the proximal femoral screw insertion site occurred and was managed conservatively. |
| Feb 2022 | Left femoral fracture fixation and  right distal femoral fracture fixation. | The left femoral incomplete shaft fracture was treated surgically. The following day, severe right knee pain developed, and a new right distal femoral fracture was diagnosed, likely related to traction-table positioning in the setting of severe skeletal fragility. | Low-dose asfotase alfa | Intramedullary nailing of the left femur was performed, followed by distal femoral plate fixation for the right distal femoral fracture. |
| May 2022 | Adjunctive anabolic therapy for delayed healing. | Persistent skeletal fragility and delayed union after bilateral femoral fixation. | Low-dose asfotase alfa + daily teriparatide 20 μg | Bone turnover markers increased markedly after teriparatide initiation |
| Oct 2022 | Six-month follow-up | Healing was observed at the right subtrochanteric, right distal femoral, and left femoral fracture sites. | Teriparatide was discontinued after 6 months; low-dose asfotase alfa was continued. | The patient achieved fracture union and symptomatic improvement. |
| Aug 2025 | Latest follow-up | Radiographs showed complete union of the right subtrochanteric, right distal femoral, and left femoral fractures. |  | The patient remained pain-free and walked independently without support. |
